# Multi-omics integration and machine learning identify NPC2 as a prognostic and treatment-responsive regulator in lung adenocarcinoma

**DOI:** 10.3389/fimmu.2025.1697560

**Published:** 2026-01-16

**Authors:** Ang Li, Ping Cui, Lili Liu, Jiawei Liu, Xianlei Zhou, Wenlong Wu, Zimo Yan, Yi Guan, Hongmei Zhang

**Affiliations:** 1 School of Public Health, North China University of Science and Technology, Tangshan, China; 2 Department of Public Health, Jining Medical University, Jining, Shandong, China; 3 Healthcare Associated Infection Control Department, Affiliated Hospital of Jining Medical University, Jining, China; 4 Department of Immunology, Tianjin Medical University Cancer Institute and Hospital, Tianjin, China; 5 College of Life Science, North China University of Science and Technology, Tangshan, China; 6 School of Clinical Medicine, North China University of Science and Technology, Tangshan, China

**Keywords:** innate immunity, lung adenocarcinoma, machine learning, multi-omics, NPC2

## Abstract

**Background:**

This study aims to define a novel molecular subtype of LUAD by integrating multiple omics data. Additionally, we develop and validate an Artificial Intelligence Derived Prognostic Index (AIDPI) that predicts the prognosis of LUAD patients, identifies potential therapeutic targets.

**Methods:**

This study employed ten clustering algorithms from the R package “MOVICS” to integrate multi-omics data of LUAD sourced from TCGA database for molecular typing. Subsequently, an Artificial Intelligence Derived Prognostic Index (AIDPI) was constructed as the most effective indicator for predicting the overall survival rate of LUAD patients. The biological functions and mechanisms of NPC2 in lung adenocarcinoma were elucidated through both *in vitro* and *in vivo* experiments, which included CCK-8 assays, colony formation assays, flow cytometry, Transwell assays, and xenograft tumor models. Additionally, the impact of NPC2 on Ribociclib sensitivity was investigated through drug correlation analysis and molecular docking, while the predictive value of NPC2 regarding immunotherapy benefits was validated using the immune cell infiltration analysis.

**Results:**

Through multi-omics clustering, we identified two subtypes of lung adenocarcinoma associated with prognosis, with the CS1 subtype exhibiting the most favorable prognostic outcomes. The low AIDPI group exhibited a more positive prognosis, accompanied by increased immune cell infiltration and activation of immune pathways. Meanwhile, NPC2 was recognized as a standalone risk factor for LUAD, with its high expression significantly improving the overall survival of LUAD patients. Functionally, the overexpression of NPC2 promotes tumorigenesis in LUAD both *in vitro* and *in vivo*. Mechanistically, the upregulation of NPC2 expression inhibits the progression of LUAD by suppressing the PI3K/AKT signaling pathway. Our study also demonstrated that high NPC2 expression is positively correlated with Ribociclib sensitivity, as confirmed by *in vitro* experiments. Furthermore, NPC2 expression is positively correlated with ImmuneScore, and may serve as a predictive indicator for the efficacy of immune checkpoint inhibitor (ICI) therapy.

**Conclusion:**

The comprehensive analysis of multiple omics data significantly enhances the molecular classification of lung adenocarcinoma. Furthermore, AIDPI is a potential biomarker that predicts the prognosis of LUAD patients. NPC2 inhibits the progression of LUAD by suppressing the PI3K/AKT signaling pathway and enhancing the chemotherapy sensitivity to Ribociclib.

## 1 Introduction

Adenocarcinoma of the lung represents the most prevalent pathological type among non-small cell lung cancers (1), with over 60% of patients diagnosed at stage III or even stage IV. Despite advancements in diagnostic techniques and treatment modalities, the inherent high heterogeneity and drug resistance associated with lung cancer contribute to a dismal 5-year survival rate of less than 15% (2, 3). Recently, candidate biomarkers for the clinical diagnosis of patients have emerged, including PD-L1 expression, TMB, neoantigen burden, mismatch repair defects, and high microsatellite instability. However, the utility of these methods is constrained by spatiotemporal heterogeneity, moderate accuracy, and their applicability to only a small percentage of the patient population (4–6). Consequently, in the context of personalized treatment, it is imperative to identify reliable biomarkers to enhance the prognosis of LUAD and optimize the benefits of pharmacotherapy.

Recent studies indicates that alterations in epigenetics, typically induced by prolonged inflammation, can regulate various facets of cancer advancement, such as tumor occurrence, development, metastasis, and immune evasion (7–9). Toll-like receptors (TLRs) represent a significant category of pattern recognition receptors that play a critical role in bridging innate and adaptive immunity while regulating inflammatory responses (10). Recent research has shown that TLRs mediate the inflammatory response within the tumor microenvironment (TME) in a complex manner. Pathogen-associated molecular patterns (PAMPs) are identified and bound by TLRs, which then recruit and activate downstream signaling molecules, including MyD88 or TRIF. This activation leads to the engagement of transcription factors like IRF3 or NF-κB, induce the production of type I interferons and inflammatory mediators, and participate in immune inflammatory responses (11, 12). As the most extensively studied pattern recognition receptors, TLR activation promotes dendritic cells maturation and cytokine production, recruits CD8^+^ T cells, and activates specific T cell immune responses, thereby inhibiting tumor occurrence and progression (13, 14). Previous reports have indicated that the regulation of TLR activation is closely associated with the reactivation of tumor-infiltrating lymphocytes’ cytotoxic activity (15, 16), and when combined with chemotherapy or immunotherapy, it can also inhibit tumor growth (17). Therefore, an in-depth exploration of the impact of the TLR pathway on tumor cells and its potential clinical significance is of great scientific importance.

The high intertumor and intratumor heterogeneity of lung adenocarcinoma can result in significantly different clinical outcomes for patients at the same stage (18, 19). In clinical settings, the processes of making decisions, managing treatments, and conducting follow-ups continue to depend on conventional TNM staging systems that are based on anatomical features, which do not take molecular biological features into account. A suitable biomarker ought to demonstrate consistent expression both internally and externally within tumor tissues to operate efficiently for all patients. Consequently, multi-gene expression analysis may represent an encouraging strategy to tackle this variability (20). Recent advancements in multi-omics integration and machine learning have revolutionized the molecular stratification of cancers, offering new avenues for prognostic modeling and therapeutic target identification. For instance, Chu et al. employed ten multi-omics clustering algorithms to identify consensus molecular subtypes in muscle-invasive urothelial cancer, demonstrating that integrative approaches enhance prognostic accuracy and reveal subtype-specific therapeutic vulnerabilities (21). Similarly, in lung adenocarcinoma and clear cell renal cell carcinoma, similar frameworks were used to construct molecular subtypes or prognostic models, effectively predicting the prognosis and immune therapy response of patients (22, 23). These studies underscore the potential of multi-omics consensus clustering and machine learning in uncovering biologically relevant subtypes and prognostic biomarkers.

This study developed integrated common subtypes of LUAD, combining mRNA transcriptome, genomic mutation, and epigenetic DNA methylation data of Toll-like receptor pathway genes. Subsequently, various machine learning algorithms were utilized to construct the Artificial Intelligence Derived Prognostic Index (AIDPI) and identify the key gene NPC2. The NPC2 protein, a small glycoprotein that is soluble, plays a crucial role in binding free cholesterol and regulates its transport and homeostasis within cells (24). Further exploration was conducted on the biological roles and molecular processes of NPC2 in LUAD, offering a foundational framework and new directions for the prevention and treatment of this disease.

## 2 Material and methods

### 2.1 Acquisition and preprocessing of multi omics data

This study collected comprehensive mRNA transcriptome expression profiles, DNA methylation status, somatic mutation profiles, and clinical characteristic data of lung adenocarcinoma patients from the Cancer Genome Atlas (TCGA) database. The gene symbols and names of mRNA were annotated using the GENCODE 27 annotation file. The beta values of DNA methylation were obtained from the Illumina DNA Methylation 450 platform via the UCSC Xena website. Somatic mutations were acquired through cBioPortal (https://www.cbioportal.org/) and processed using the maftools software package. After merging all available omics and clinical features, a total of 429 lung adenocarcinoma patients were included in this study for further analysis.

### 2.2 Acquisition and preprocessing of external validation data

To validate the new findings from multiple omics cohorts, this study selected three external Gene Expression Omnibus (GEO) cohorts, namely GSE72094, GSE13213, and GSE50081, from the GEO database. The mRNA expression matrices and clinical data were obtained from these cohorts. To ensure accuracy and consistency, probes corresponding to multiple molecules were initially removed, retaining only the probe data with the highest signal intensity for each molecule. All chip expression profile data underwent background correction using the limma software package, followed by log2 transformation and normalization. **Table 1** presents a detailed list of the basic feature information of the dataset.

**Table 1:** Basic information of GEO LUAD datasets.

GEO datasets	Platform	Disease	Sample		Years	Country
			Control	Case		
GSE72094	GPL15048	LUAD	0	442	2018	United States
GSE13213	GPL6480	LUAD	0	117	2019	Japan
GSE50081	GPL570	LUAD	0	127	2019	Canada

### 2.3 Multi omics comprehensive clustering analysis

The core functions of the MOVICS software package consist of three modules: the GET module is responsible for the comprehensive clustering of multiple omics data to identify molecular subtypes; the COMP module is utilized for multidimensional comparisons of features across different subtypes; and the RUN module performs tag recognition and subtype verification tasks. This study employed the “MOVICS” multi-omics integrated analysis R software package to integrate various omics data, including the mRNA transcriptome expression profile of the Toll like receptor signaling pathway gene set, all methylation probe data obtained the UCSC platform (Illumina DNA Methylation 450 chip), and somatic mutation profiles (missense, nonsense, splice site mutations, etc.) from whole exome or whole genome sequencing data, to identify molecular subtypes of lung adenocarcinoma. Initially, the TPM expression data was assessed using univariate Cox regression analysis after log2 transformation, with a screening threshold set at *P* < 0.05. To ascertain the optimal number of subtypes, the “getClustNum” function was employed, in conjunction with multiple omics data, to calculate the clustering prediction index and gap statistic. Subsequently, based on the identified subtypes, patients were classified using ten clustering algorithms, including iClusterBayes, moCluster, CIMLR, IntNMF, ConsensusClustering, COCA, NEMO, PINSPlus, and SNF, with LRA as an additional method. By employing a consistency ensemble algorithm, the clustering results were integrated to ensure that the same sample was categorized consistently across different algorithms. Finally, to account for potential discrepancies in omics data, the “getConsensus MOIC” function was utilized to calculate the consistency matrix, quantify the robustness of sample clustering, screen for highly robust subtypes, and evaluate the similarity of samples within subtypes through contour scoring.

### 2.4 Establish a comprehensive machine learning driven prognostic model

Based on transcriptome data from The Cancer Genome Atlas Lung Adenocarcinoma (TCGA- LUAD), the “limma” package was utilized to analyze differentially expressed genes (DEGs) between normal and tumor tissues, as well as among different molecular subtypes. The screening criteria were established at |log2FC| > 2 and FDR < 0.05. Additionally, the “VennDiagram” package was employed to visualize the intersecting DEGs between the two groups. Subsequently, univariate Cox regression analysis was conducted to identify genes with potential prognostic significance for further investigation. This study utilized TCGA-LUAD data as the model training set and selected three independent lung adenocarcinoma cohorts (GSE72094, GSE13213, and GSE50081) from the GEO database as external validation sets. To construct the optimal prognostic model, we integrated ten machine learning methods, including CoxBoost, stepwise Cox regression, Lasso regression, ridge regression, elastic net, support vector machine, generalized additive model, supervised principal component analysis, partial least squares regression, and random survival forest, resulting in a total of 101 algorithm combinations. Each algorithm combination model was validated on both internal and external validation sets, and its robustness and clinical applicability were assessed using the concordance index (C-index). According to the median AIDPI for each group, patients with lung adenocarcinoma were divided into high AIDPI and low AIDPI categories. The prognostic value was analyzed through Kaplan-Meier curves, and Receiver Operating Characteristic (ROC) curves were created to evaluate the prognostic efficacy of AIDPI.

### 2.5 Gene set variation analysis and gene set enrichment analysis

The estimation of a collection of 50 hallmark gene sets (h.all.v7.5.symbols.gmt) obtained from the MsigDB database was performed using GSVA and GSEA. The “ComplexHeatmap” R package was utilized to visualize significant pathways across various groups.

### 2.6 TME analysis

To assess the abundance of cells within the tumor microenvironment (TME), we analyzed the expression of NPC2 in individual cells using the TISCH2 database (http://tisch.comp-genomics.org/home/). Then, we evaluated the presence of 24 different immune cell types in the TME using Gene Set Variation Analysis (GSVA). An analysis of the distribution of immune checkpoints was carried out, along with the determination of immune and stromal scores for tumor tissues through the “ESTIMATE” package in R. These correlation results were visualized using heatmaps.

### 2.7 Survival analysis

This study obtained three clinical prognostic endpoint data from the TCGA database: overall survival (OS), progression free interval (PFI), and disease-specific survival (DSS), to comprehensively evaluate the predictive performance of the model. (1) OS: refers to the time from the diagnosis of a patient to death for any reason. (2) PFI: refers to the time from diagnosis to the first occurrence of any of the following events (primary tumor progression, local recurrence, distant metastasis, or death from any cause). (3) DSS: refers to the time from diagnosis to direct death due to the disease. Due to varying definitions of endpoints, there are inherent differences in sample sizes and event counts across the analyzed cohorts. Survival analysis was visualized using the Kaplan-Meier method, and between-group comparisons were performed with the log-rank test. All analyses were carried out using the “Survival” package and the “Survminer” package in R software.

### 2.8 Quantify the immunotherapy response predictor

The Tumor Immune Dysfunction and Exclusion (TIDE) framework serves as a tool to assess the likelihood of tumor immune evasion by analyzing tumor gene expression data. We analyzed mRNA-seq data from the TCGA-LUAD, GSE50081, GSE72094, and GSE13213 employing the TIDE algorithms to forecast how patients might respond to Immune-Checkpoint Blocker (ICB) therapy.

### 2.9 Construction of nomogram

Nomogram plots were constructed by combining the NPC2 expression and various clinical data. In the nomogram graph, every factor was associated with a specific score, which contributed to the cumulative score. The overall scores were then utilized to predict the 1-year, 3-year, and 5-year overall survival (OS) rates; a higher score correlated with a decreased survival rate.

### 2.10 Cell culture

The human LUAD cell lines A549 and PC9 were provided by Pricella Biotechnology (Wuhan, China) and maintained in RPMI 1640 supplemented with 10% FBS and 0.1 mg/mL penicillin-streptomycin under regular conditions (5% CO2, 37 °C).

### 2.11 CCK-8 assay

Cell growth was assessed with a CCK-8 kit (Dojindo, Kumamoto, Japan) based on the manufacturer’s recommendations. A549 and PC9 cells were seeded into 96-well plates, and incubated for 24, 48, and 72 hours. Next, cells were incubated for 1h after adding with 10µL CCK-8 solution. Eventually, the OD450nm was measured using a microplate reader (BioTek, Winooski, VT, USA).

### 2.12 Clone formation

1×10^3^ cells were plated into a 35 mm dish and maintained at 37°C and 5% CO2 for 14 days or observed under microscope when the number of cells in a single cell mass exceeded 50. Afterwards, the cells were fixed with 4% paraformaldehyde and stain with 0.1% crystal violet (Solarbio, Beijing, China) to count colony numbers.

### 2.13 Cell migration and invasion assay

Cell migration and invasion was evaluated by a transwell assay. Tumor cells were inoculated into the upper chamber coated with or without Matrigel (Corning Inc., Corning, NY, USA). The lower chamber was filled with RPMI 1640 and 10%FBS. After one day’s incubation, cells in the lower chamber were stained with 0.2% crystal violet for 20min in the dark, and stained cells were captured under a microscope (Olympus, Tokyo, Japan).

### 2.14 Real-time fluorescence quantitative PCR

RNA was extracted from tissues or cells with Trizol reagent (Thermo Fisher Scientific, MA, USA) and RNA reverse transcription was performed using RevertAid First Strand cDNA Synthesis Kit (Thermo Fisher Scientific, MA, USA). After this step, use SYBRGreen Mix (Mei5 Biotechnology, Beijing, CHN) for RT-qPCR. The 2^-△△ct^ method was used, GAPDH was applied as an internal control for normalizing. The primer sequences utilized in the experiment are shown in **Supplementary Table 1**.

### 2.15 Apoptosis assay

FITC-Annexin V and propidium iodide were used for double staining in accordance with the Annexin V-FITC/PI Cell Apoptosis Detection Kit (Servicebio, Wuhan, China) manufacturer’s instructions, followed by flow cytometry. Flow cytometry data were imported and analysed using the flowCore and flowGate packages.

### 2.16 Western blot analysis

Lysed cells with RIPA buffer (Thermo Fisher Scientific, MA, USA), and the concentration of total protein was measured by BCA kit (Solarbio, Beijing, China). It was electrophoresed with 10% SDS-PAGE gel, then transferred to nitrocellulose film for 70 minutes at 200mA, sealed with 5% skim milk powder for 1 hour, then incubated overnight at 4°C in primary antibody, washed with TBST and incubated in the HRP-conjugated second antibody at room temperature for 1 hour, then washed with TBST for three times, each time for 10 minutes, and Immunoreactive proteins were detected by enhanced chemiluminescence reagent (Pierce, Thermo Scientific) using a Bioimaging System. The antibodies used are as follows: Bax (ab32503), Bcl-2 (ab182858), E-Cadherin (ab231303), N-Cadherin (ab245117), PI3K (ab191606), p-AKT (phospho T308) (ab38449) and p-PI3K (phospho Y464) (ab138364) were purchased from Abcam (Eugene, USA). *β*-actin was used as a control.

### 2.17 Immunohistochemical analysis

Paraffin was used to embed tissue samples, and sliced thinly colored with hematopoietic and eosin dyes (C0105M, Beyotime, China). For immunohistochemistry, the sections were incubated with primary antibodies at 4°C overnight. Subsequently, the sample underwent washing with PBS and incubated with the secondary antibody at 37 °C for 2h. After being washed and dehydrated with gradient ethanol and treated with xylene in turn before being prepared for microscopic observation. The mouse antibodies used are as follows: Ki-67 (GB14102) antibodies were purchased from Servicebio (Wuhan, China).

### 2.18 Xenograft models

Male BALB/c nude mice at four weeks of age were randomly assigned to two groups, with six mice in each group. A549 cells (3×10^6^/0.2ml PBS) stably transfected with NPC2 and empty vector were subcutaneously injected into nude mice to establish xenograft model. Xenografts were examined every 3 days with digital calipers. After 23 days, the mice were sacrificed and stripped the tumors for further analysis. All mice were bought from SPF Biotechnology (Beijing, China) and used in accordance with the agreement approved by the Animal Ethics and Welfare Committee of North China University of Science and Technology (2025-SY-3048).

### 2.19 Drug sensitivity analysis

The Genomics of Drug Sensitivity in Cancer (GDSC) database comprises a vast collection of genomic datasets and drug sensitivity information. We utilized the “pRRopheticPredict” R package (version 0.5) to construct statistical models leveraging gene expression data derived from a large array of cancer cell lines. Subsequently, we applied these models to the gene expression data of the target samples to assess the variations in IC50 values of 198 drugs between the different NPC2 expression.

### 2.20 Molecular docking

The interaction between Ribociclib and NPC2 was examined through molecular docking techniques. The 3D structure of Ribociclib was obtained from the PubChem database (https://pubchem.ncbi.nlm.nih.gov/) and subsequently converted into PDB format utilizing Open Babel 2.3.2 software. The 3D structure of the NPC2 protein was retrieved from the protein database and downloaded in PDB format. The ligand and water molecules were eliminated using this software. Next, PYMOL 2.3.4 was employed to modify the NPC2 protein by adding hydrogen atoms, balancing the charge, and performing other necessary operations through Auto DockTools, which produced PDB files. These were then converted into pdbqt format for subsequent processing. The docking simulations were conducted using AutoDock Vina 1.1.2 software, and the results were visualized with PYMOL.

### 2.21 Statistical analysis

The data are presented as mean ± standard deviation (SD). To assess the differential expression between the two groups, the Student’s t test was utilized. Survival analysis was conducted using the Kaplan-Meier method and assessed with the log-rank test. All statistical evaluations were carried out with R software (version 4.2.3), SPSS 23.0 (Chicago, IL), and GraphPad Prism 6 (La Jolla, CA, USA). A *P*-value of less than 0.05 was deemed statistically significant.

## 3 Results

### 3.1 Multi omics integrated consensus subtypes of lung adenocarcinoma

**Figure 1:**
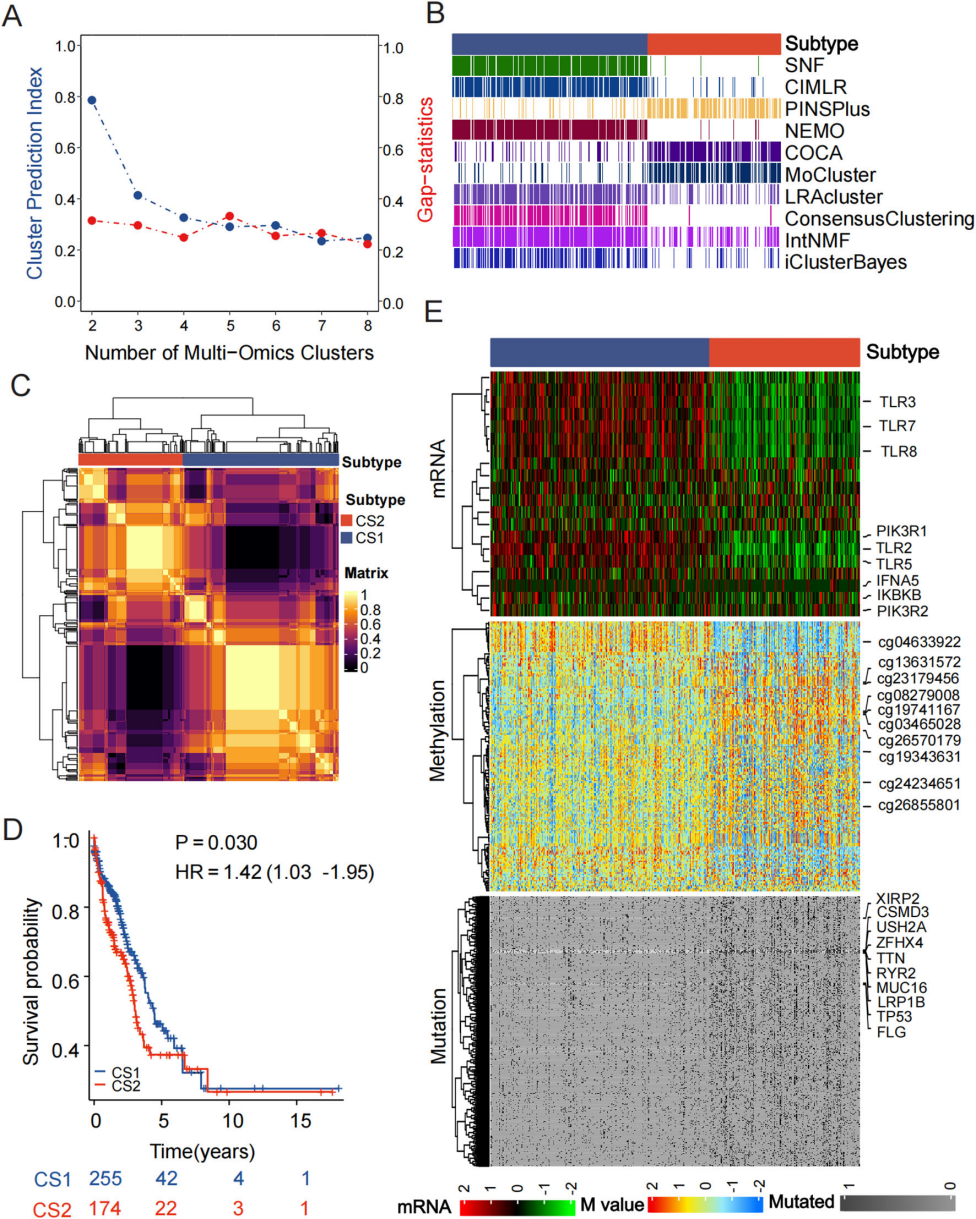
The multiomics integrative consensus subtypes of LUAD. **(A)** Cluster prediction indicators and Gap statistics predict the optimal number of clusters for multi omics clustering. **(B)** Clustering of LUAD patients through 10 cutting-edge multiomics clustering methods. **(C)** A consensus clustering matrix showcasing three new prognostic subtypes derived from the ten algorithms. **(D)** Varied survival outcomes observed between the two subtypes. **(E)** Comprehensive heatmap of consensus ensemble subtypes.

Based on the Toll like receptor pathway, we found that according to the results of CPI analysis and gap statistical analysis, the highest average statistical value of subtype number was defined as the two clustering results, and the scores of the two analysis methods were more similar (**Figure 1A**). Furthermore, we utilized 10 multi omics ensemble clustering algorithms to classify patients (**Figure 1B**), and aggregated them through consistent classification to obtain a more stable clustering result (**Figure 1C**). Finally, two new molecular subtypes of lung adenocarcinoma were named CS1 and CS2, respectively. Patients with the CS2 subtype experienced the poorest clinical outcomes, in contrast to those with the CS1 subtype, who exhibited the longest overall survival times (*P* < 0.03, **Figure 1D**). Utilizing multi-omics data from the TCGA-LUAD cohort, the varying distributions of mRNA expression levels, DNA methylation at CpG sites, and mutant genes were subsequently illustrated through a consensus ensemble approach (**Figure 1E**).

### 3.2 Development and validation of the AIDPI

**Figure 2:**
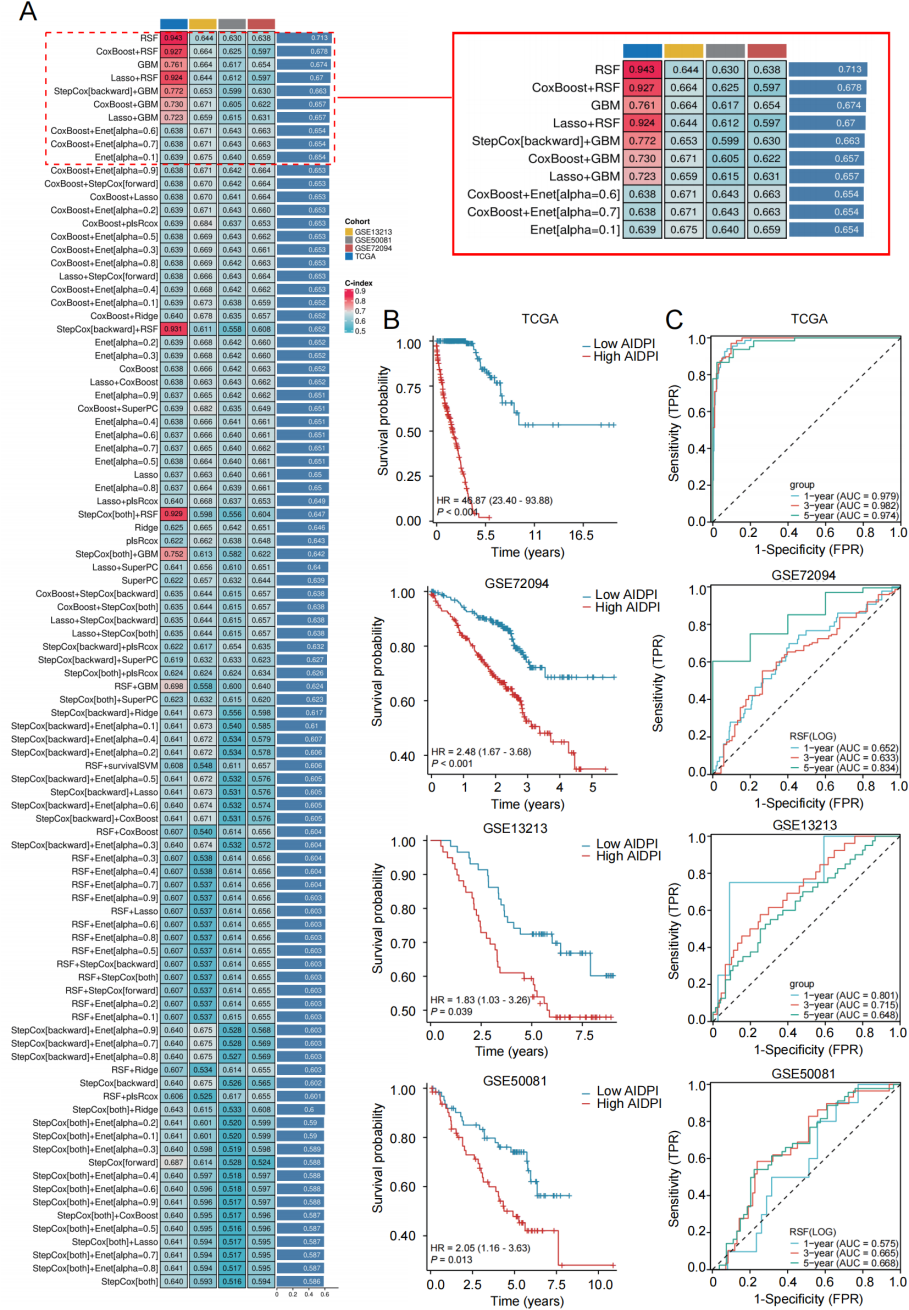
Development and validation of the AIDPI. **(A)** C-indices obtained from different combinations of machine-learning algorithms across three cohorts. **(B)** Kaplan-Meier survival analyses of the AIDPI across multiple cohorts. **(C)** Time-dependent ROC curve assessments of the AIDPI performed in several cohorts.

We developed the AIDPI following the schematic in **Supplementary Figure S1A**. Initially, TCGA-LUAD cohort was employed as a training set. Meanwhile, GSE72094, GSE13213 and GSE50081 served as our validation set. Based on the expression profiles of 235 CS1 vs CS2 difference gene expression, univariate Cox analysis identified 47 prognostic mRNA and shared between the training and validation sets (**Supplementary Figures S1B-C**). The CPGs were incorporated into a machine-learning framework to develop 101 different prediction models using the TCGA-LUAD dataset. Subsequently, the C-index of each model was computed across all validation datasets. The model created through the combination of RSF was selected as the best model since it had the highest average C-index of 0.713 (**Figure 2A**). Using this model, we computed the AIDPI for each patient within various cohorts. According to the optimal threshold of the AIDPI established from the training set, LUAD patients were divided into low AIDPI and high AIDPI groups. Furthermore, to more rigorously validate our model, we assessed the predictive performance of AIDPS in the validation cohorts. Kaplan-Meier survival analysis indicated that the low AIDPS group experienced significantly longer overall survival (OS) in the three external validation cohorts (log-rank *P* < 0.001 in the GSE72094, *P* = 0.039 in the GSE13213, *P* = 0.013 in the GSE50081) (**Figure 2B**). The ROC analyses revealed that the areas under the ROC curves (AUCs) in the training set (TCGA) were 0.979,0.982 and 0.974 for 1-, 3- and 5-year OS, respectively. The AUCs in the GSE72094 validation set were 0.652, 0.633 and 0.834, GSE13213 validation set were 0.801, 0.715 and 0.648, GSE50081 validation set were 0.575, 0.665 and 0.668 (**Figure 2C**). Overall, the AIDPS demonstrated a reliable and precise ability to forecast the outcomes for patients with PACA, indicating that it could emerge as a valuable resource for clinical application.

### 3.3 Prediction of clinical features and immune processes based on AIDPI

**Figure 3:**
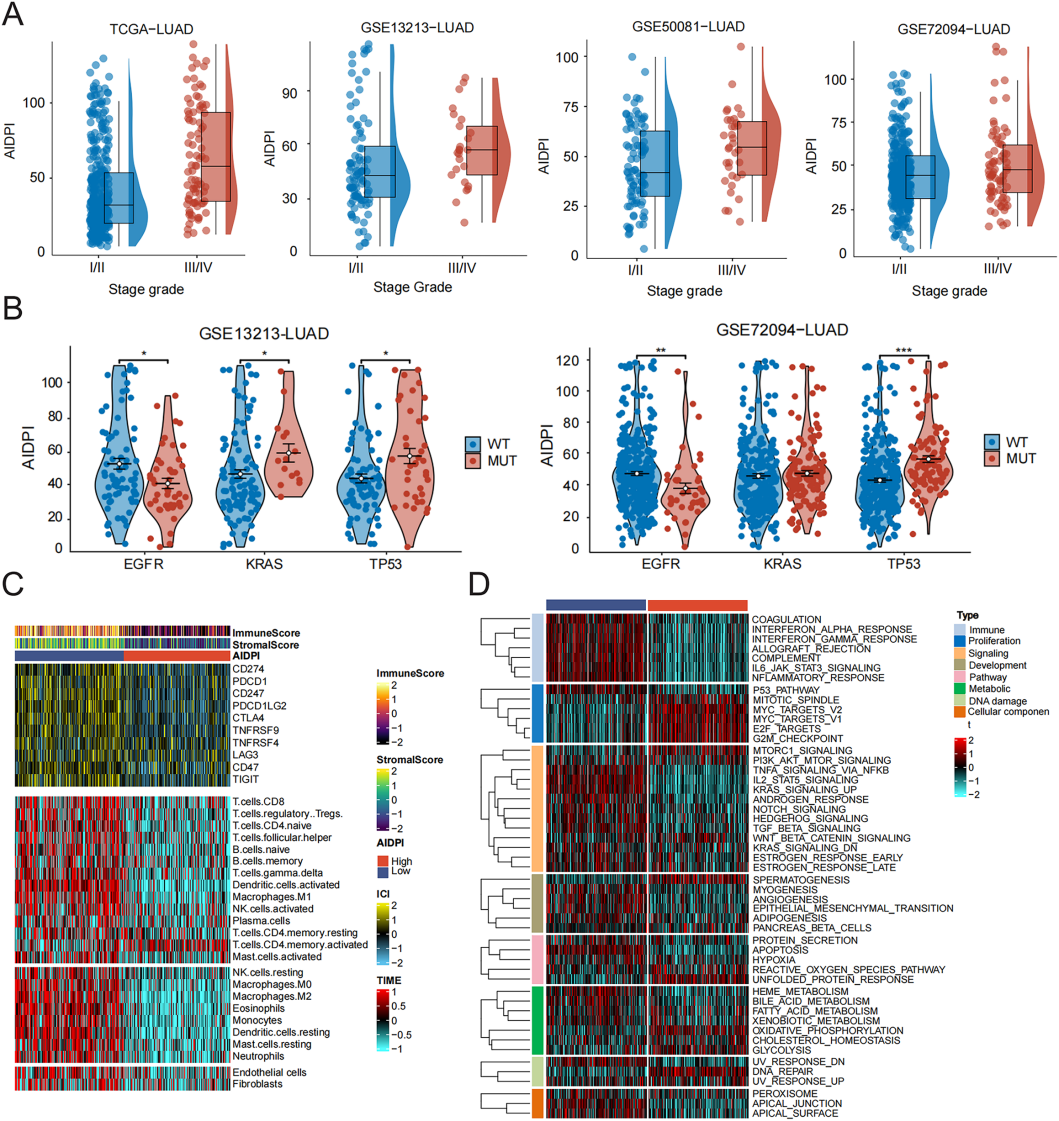
Prediction of clinical features and immune processes based on AIDPI. **(A)** AIDPI distributions across varying pathological staging in the indicated cohorts. **(B)** AIDPI distributions across GFR, KRAS, and TP53 mutations patient in the indicated cohorts. **(C)** Expression of immune checkpoints and the enrichment of immune cell infiltration within the TCGA cohort. **(D)** Signal pathway enrichment analysis of 50 hallmark gene sets.

Next, we will compare the clinical information and pathological feature distribution of LUAD patients in high- and low- AIDPI groups and sought to elucidate relationships between these factors and the AIDPI. Result revealed significant associations between high- and low- AIDPI groups and parameters such as T stage (*χ*^2^ = 13.011, *P* = 0.001), M stage (*χ*^2^ = 4.798, *P* = 0.028), pathologic stage (*χ*^2^ = 10.605, *P* = 0.001) and radiotherapy (*χ*^2^ = 15.501, *P* = 0.001) (**Table 2**). In summary, these results suggest that AIDPI may play an important role in tumor metastasis, disease progression, and radiotherapy response of lung adenocarcinoma, and may become a potential biomarker for evaluating the condition and treatment strategies of LUAD patients. Further analysis of multiple independent datasets (GSE13213, GSE50081, and GSE72094) revealed that AIDPI levels in patients with advanced-stage (III-IV) lung adenocarcinoma were significantly elevated compared to those in early-stage (I-II) patients (*P* < 0.05) (**Figure 3A**). Additionally, AIDPI levels were found to be lower in patients with EGFR mutations and higher in patients with TP53 mutations, as indicated by the GSE13213 and GSE72094 datasets (*P* < 0.05) (**Figure 3B**).

**Table 2:** The relationship between AIDPI level and clinicopathological characteristics of patients with lung adenocarcinoma in TCGA database.

Clinical characteristics	Number	The level of AIDPI		*χ* ^2^	*P* value
		Low (n=215)	High (n=214)		
Age				0.602	0.438
≤60	275	141 (66.5%)	134 (62.9%)		
>60	150	71 (33.5%)	79 (37.1%)		
Gender				1.735	0.188
Male	196	91 (42.9%)	105 (49.3%)		
Female	229	121 (57.1%)	108 (50.7%)		
Smoking				1.644	0.199
Yes	366	178 (84.0%)	188 (88.3%)		
No	59	34 (16.0%)	25 (11.7%)		
T Stage				3.573	0.059
T1&T2	372	192 (90.6%)	180 (84.5%)		
T3&T4	53	20 (9.4%)	33 (15.5%)		
N Stage				13.011	0.001
N0	280	156 (75.4%)	124 (58.8%)		
N1&N2&N3	138	51 (24.6%)	87 (41.2%)		
M Stage				4.798	0.028
M0	270	136 (96.5%)	134 (89.9%)		
M1	20	5 (3.5%)	15 (10.1%)		
Pathological Stage				10.605	0.001
Stage I & Stage II	283	157 (74.1%)	126 (59.2%)		
Stage III & Stage IV	142	55 (25.9%)	87 (40.8%)		
Radiotherapy				15.501	0.001
No	367	197 (92.9%)	170 (79.8%)		
Yes	58	15 (7.1%)	43 (20.2%)		
Chemotherapy				0.785	0.376
No	280	144 (67.9%)	136 (63.8%)		
Yes	145	68 (32.1%)	77 (36.2%)		

To further investigate the molecular characteristics between the high and low AIDPI groups, we selected the Hallmark gene set and quantified it using the GSVA algorithm. The results indicated that, compared to other pathways, the low AIDPI group was predominantly enriched in immune-related processes such as coagulation, complement activation, interferon-α response, and IL-6/JAK/STAT3 signaling. In contrast, the high AIDPI group exhibited enrichment mainly in proliferation-related processes, including G2M checkpoints, mitotic spindles, and E2F targets (**Figure 3D**). Given the significant differences in immune processes between the high and low AIDPI groups, we quantified the infiltration levels of immune cells in the microenvironment along with the expression of immune checkpoints. As illustrated in the **Figure 3C**, although the high AIDPI group demonstrated greater activation of CD4^+^ memory T cell infiltration, the low AIDPI group appeared to exhibit higher immune infiltration activity, characterized by the activation of plasma cells and CD8^+^ T cells, as well as substantial NK cell infiltration. Furthermore, in the low AIDPI group, anti-inflammatory immune cells such as Treg cells, follicular helper T cells, and M2 macrophages were significantly more prevalent. Notably, we observed significant differences in immune checkpoint expression between the two groups, with PD-1, PD-L1, CTLA-4, and TIGIT expression levels being markedly higher in the low AIDPI group than in the high AIDPI group. The distinct patterns of immune infiltration and immune-related gene expression among AIDPI patients in different groups suggest that the response to immunotherapy may vary.

### 3.4 NPC2 low expression in LUAD correlates with tumor progression

**Figure 4:**
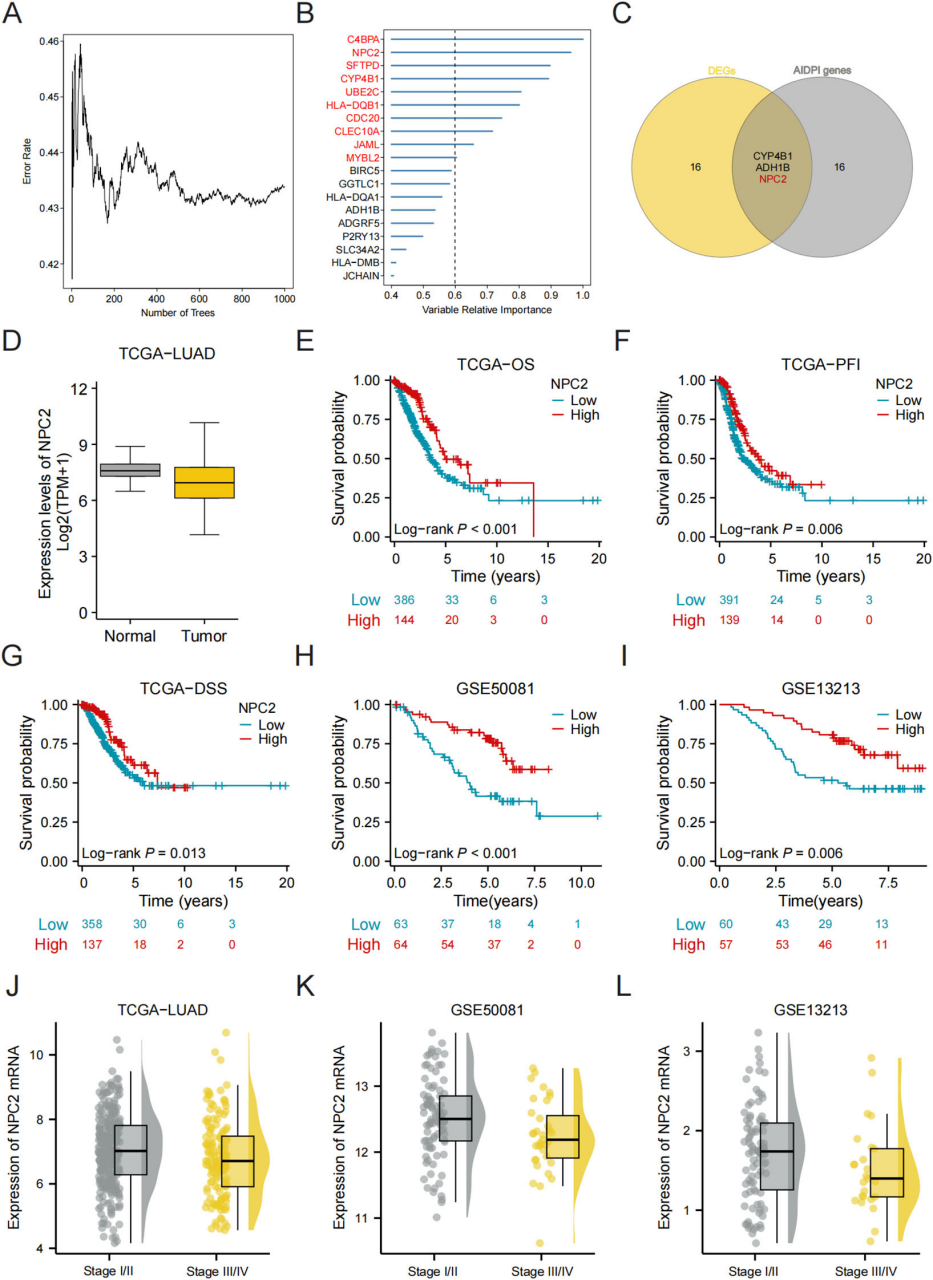
High NPC2expression is associated with good prognosis in LUAD. **(A)** The error rate related to the data according to the classification tree. **(B)** Screening of biomarkers utilizing the RF model. **(C)** A Venn diagram illustrates the common entities across the specified gene groups. **(D)** Analysis of NPC2 expression in both normal tissue and LUAD through the TCGA dataset. **(E–I)** Kaplan-Meier plots for NPC2 expression derived from the TCGA, GSE13213 and GSE50081 datasets. **(J–L)** Distribution of NPC2 expression in early and late stage LUAD patients.

We optimized the key parameters of the random forest through cross validation, particularly mtry (reducing the number of randomly selected features per tree), nodesize (increasing the minimum sample size of leaf nodes), and nsplit (limiting the number of node partitions), to enhance the regularization effect. Further assessed genes based on their significance (nrep=1000, which denotes that the Monte Carlo simulation was run for 1000 iterations; nstep=5, meaning that five steps were progressed) (**Figure 4A**). The connection between the error rate and the count of classification trees is depicted in **Figure 4B**. In the end, genes demonstrating a relative importance exceeding 0.6 were chosen as possible candidates. Through additional intersection analysis alongside differentially expressed genes from high and low AIDPI groups, we identified three overlapping genes. The expression patterns of these genes were later depicted using feature maps (**Figure 4C**). According to the canSAR database (https://cansar.ai/), only the protein encoded by CYP4B1 and NPC2 possesses a druggable structure, making it a potential target for patients with high AIDPI. In addition, there have been numerous literature reports that CYP4B1 is a prognostic biomarker and potential therapeutic target for lung adenocarcinoma, and is associated with immune cell infiltration (25, 26). Therefore, we have identified NPC2 as the main gene for further research. Our analysis of data from TCGA-LUAD reveals that the expression level of NPC2 is significantly reduced in LUAD tissues compared to normal tissues (**Figure 4D**). Follow-up research investigating the clinical results of NPC2 and LUAD within three groups (TCGA, GSE50081, and GSE13213) revealed that reduced expression of NPC2 correlates with unfavorable prognosis in patients with LUAD, supported by survival curves from various cohorts (**Figures 4E–I**). This indicates that patients in the low NPC2 group exhibit significantly lower overall survival (OS) or event-free survival rates across various LUAD cohorts. Further analysis revealed that NPC2 mRNA levels in late-stage (III-IV) lung adenocarcinoma patients were significantly higher than those in early-stage (I-II) patients (*P* < 0.05) (**Figures 4J–L**).

**Figure 5:**
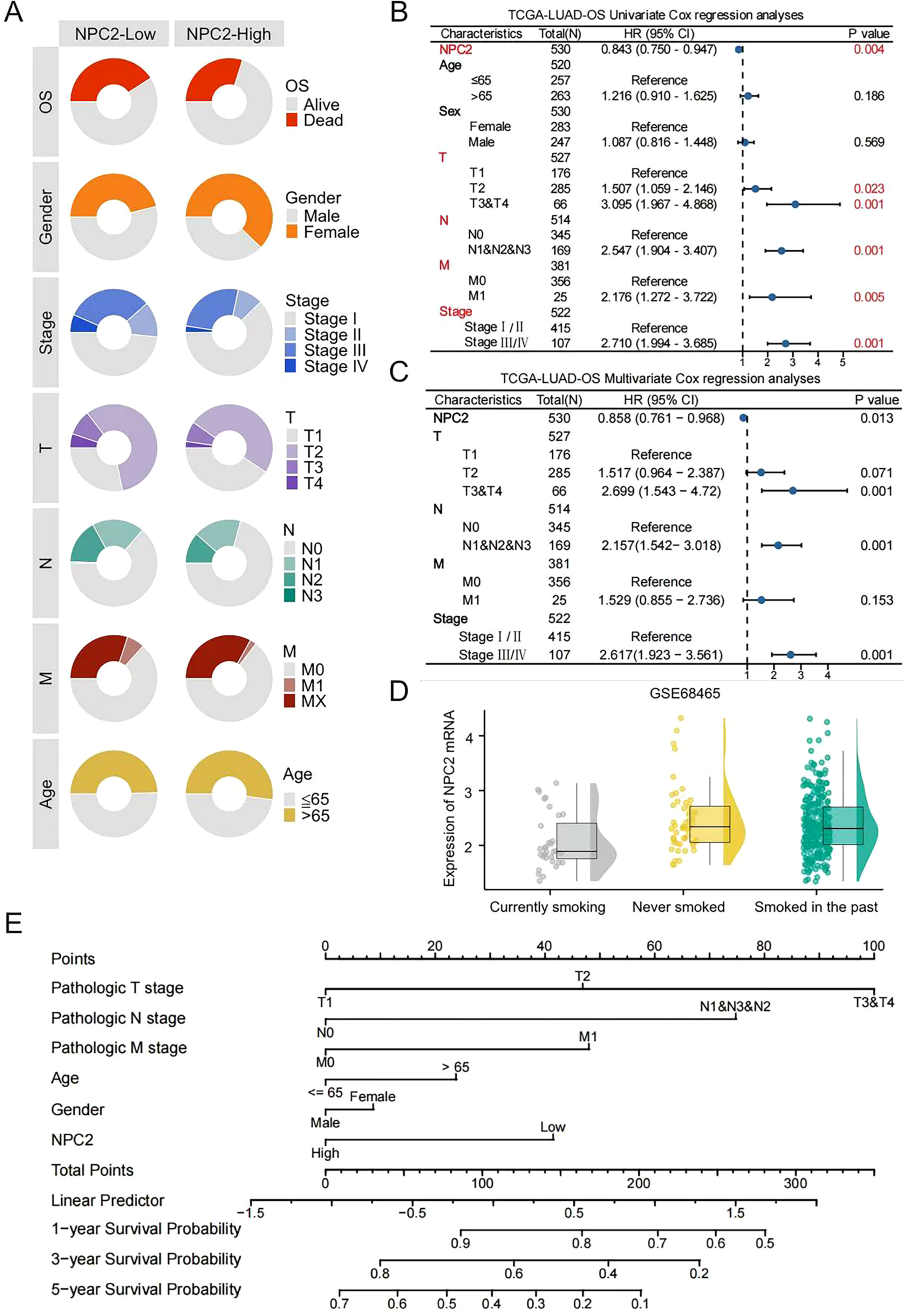
Survival prediction based on NPC2 expression and clinical features. **(A)** Distribution of clinical pathological features in different expression groups of NPC2. **(B, C)** Univariate and multivariate Cox regression analyses examining the relationship between NPC2 and clinicopathological characteristics in relation to their prognostic significance. **(D)** Distribution of NPC2 expression in LUAD patients with different smoking statuses. **(E)** Utilizing nomograms to forecast the survival rates at 1, 3, and 5 years for patients with LUAD.

Considering the acknowledged influence of various clinical factors on the outcomes for LUAD patients, we aim to elucidate the relationship between these factors and NPC2 to validate NPC2 as an independent prognostic biomarker. The multidimensional circular plot illustrating the survival status of patients with varying clinical characteristics in the NPC2 high and low expression groups demonstrates that the survival rate of patients in the NPC2 low expression group is significantly higher than that of the high expression group across several dimensions, including overall survival (OS), gender, tumor stage, primary tumor focus, lymph node metastasis, distant metastasis, and age (**Figure 5A**). Univariate and multivariate Cox regression analyses demonstrated a significant correlation between overall survival (OS) and NPC2 expression, T stage, N stage, and pathological stage in patients with lung adenocarcinoma (**Figures 5B, C**). When assessing its predictive ability in smokers, NPC2 mRNA expression was found to be lowest in smoking patients and highest in non-smoking lung adenocarcinoma patients (**Figure 5D**). Based on these findings, we constructed a nomogram model to estimate the effect of NPC2 expression on the OS of lung adenocarcinoma patients at 1, 3, and 5 years. This model incorporates age, gender, TNM staging, and NPC2 mRNA expression, as illustrated in **Figure 5E**. Overall, our results indicate that the column chart model based on NPC2 demonstrates strong predictive performance for the prognosis of LUAD patients.

### 3.5 NPC2 overexpression impedes LUAD *in vitro* and *in vivo*

**Figure 6:**
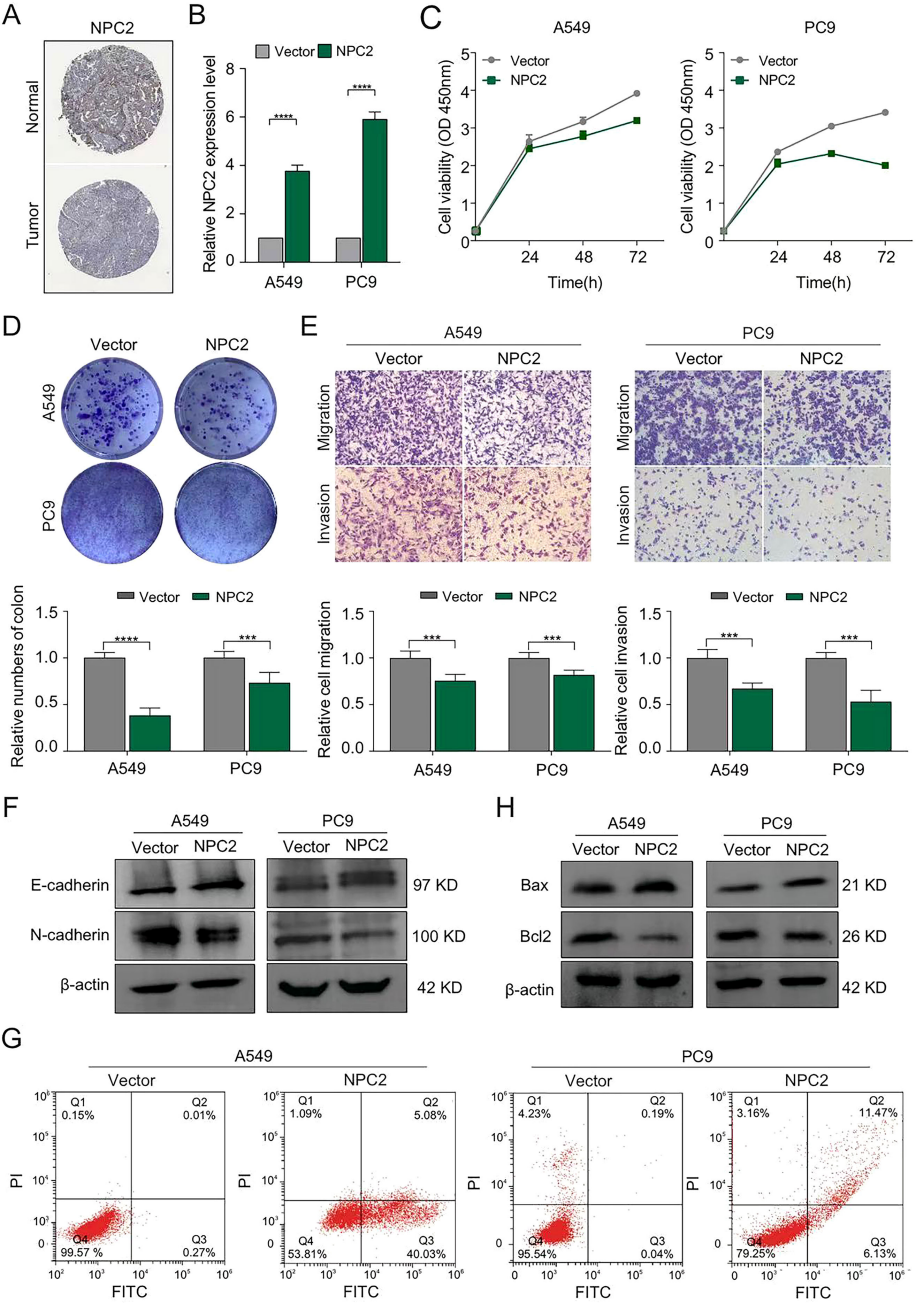
NPC2 inhibits tumor growth and metastasis of lung adenocarcinoma. **(A)** Analysis of protein expression differences of NPC2 in lung adenocarcinoma tissue and normal tissue through HPA database. **(B)** qRT-PCR detection of overexpression efficiency of NPC2. **(C)** CCK-8 assay in LUAD cells stably up-regulating NPC2 on cell viability. **(D)** Colony formation assays in LUAD cells stably up-regulating NPC2. **(E)** The transwell assays were utilized to assess the migration and invasion of the specified LUAD cells. **(F)** The epithelial mesenchymal transition related proteins E-cad and N-cad were detected by western blot following NPC2 overexpression. **(G)** Apoptosis related proteins were detected after NPC2 overexpression. **(H)** The analysis of cell apoptosis resulting from the overexpression of NPC2 was conducted using flow cytometric assays. The data are the means ± SEM. ***P < 0.001, ****P < 0.0001.

The Human Protein Atlas database indicates low NPC2 protein abundance in LUAD tissues by representative immunohistochemical images (**Figure 6A**). To clarify the role of NPC2 in cell biological behaviors, we established stable A549 and PC9 cell through lentiviral-mediated NPC2 overexpression or control with verified the overexpression efficiency (**Figure 6B**). Function assays using these cell lines revealed that NPC2 overexpression significantly suppressed colony formation and proliferation of LUAD cells (**Figures 6C, D**). Moreover to gain insights into the effect of NPC2 on LUAD metastasis, transwell migration/invasion assays were performed (**Figure 6E**). The results showed that NPC2 suppressed LUAD cell metastasis *in vitro*. The transition from epithelial to mesenchymal cells (EMT) is an essential characteristic associated with the invasion and metastasis of tumors. Consequently, western blot assays were employed to analyze markers related to EMT. The findings indicated that the overexpression of NPC2 enhanced the levels of E-cadherin while reducing the levels of N-cadherin (**Figure 6F**). To elucidate the mechanism underlying NPC2 induction of LUAD cell proliferation, cell apoptosis were further detected by flow cytometry. The results verified that NPC2 overexpression increased the rate of apoptotic cells (**Figure 6G**). Consistent with the flow cytometry results,NPC2 overexpression significantly downregulated apoptosis inhibitory protein Bcl-2, and upregulated the expression of proapoptotic proteins Bax (**Figure 6H**).Taken together, these data provided evidences that NPC2 suppressed LUAD cell proliferation, promoted cell apoptosis, and cell metastasis *in vitro*.

### 3.6 NPC2 overexpression inhibits PI3K/AKT pathway and suppresses *in vivo* tumor growth in LUAD

**Figure 7:**
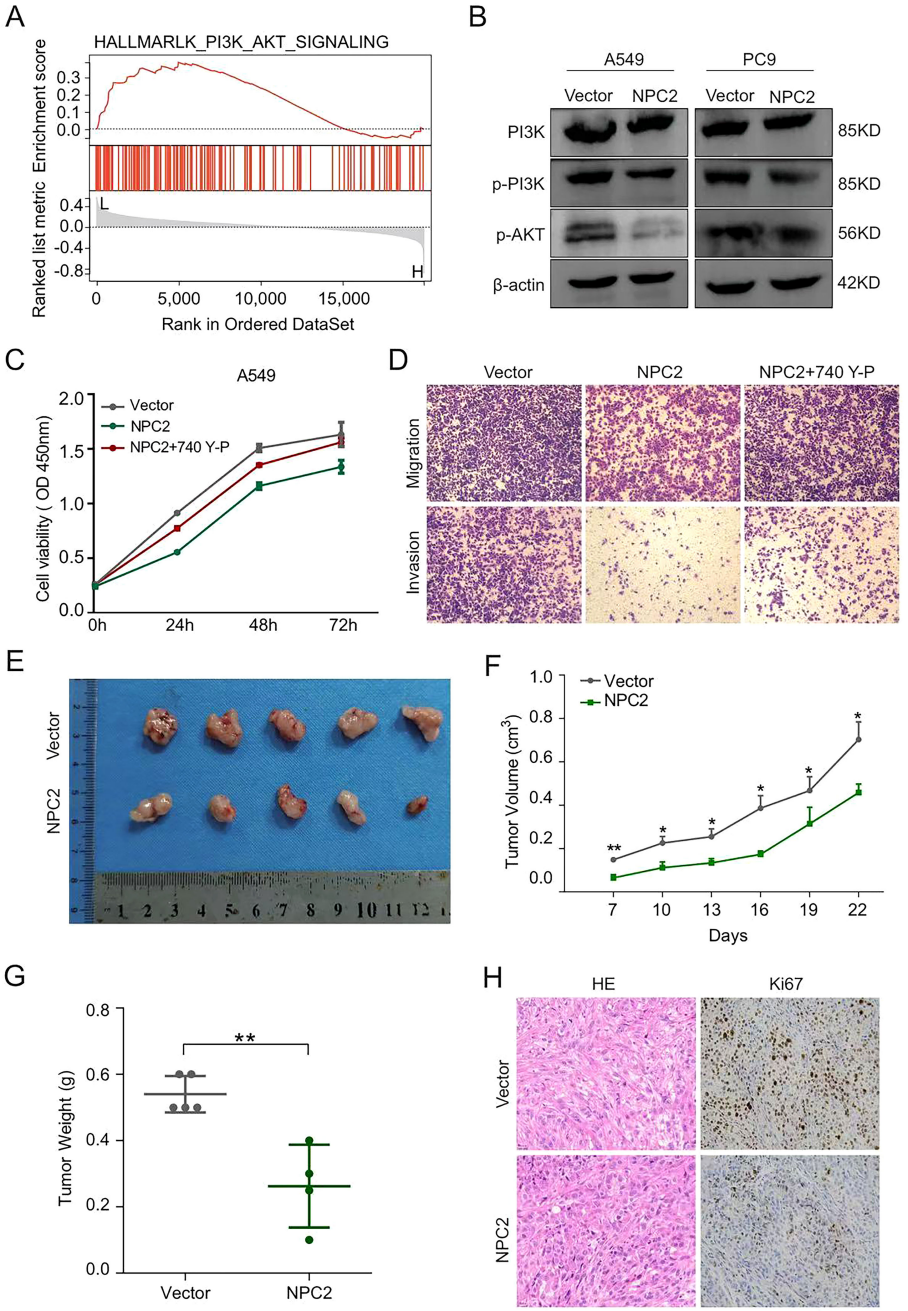
NPC2 overexpression inhibits PI3K/AKT pathway and suppresses *in vivo* tumor growth in LUAD. **(A)** GSEA results were illustrated to demonstrate the association of NPC2 with the PI3K/AKT signaling pathway. **(B)** Evaluate the expression levels of crucial proteins within the PI3K/AKT pathway in LUAD cells. **(C)** CCK-8 assays were used to observe the growth of LUAD cells. **(D)** Cell migration and invasion ability were observed using the transwell assay. **(E)** Representative image of tumors formed in nude mice. **(F)** Growth curves showing the subcutaneous tumor volume across various groups. **(G)** Subcutaneous weight of tumor in nude mice. **(H)** Representative images for IHC staining of tumor samples from the different groups. **P < 0.01.

In order to uncover intracellular pathways that could facilitate the influence of NPC2 within LUAD cells, we conducted a bioinformatics analysis of related genes derived from the TCGA-LUAD dataset. Hallmark pathway analysis showed that NPC2 was linked to the PI3K/AKT pathway (**Figure 7A**). Western blot analysis of A549 and PC9 cells demonstrated a significant decrease in the expression levels of the active, phosphorylated (p) forms of AKT and PI3K proteins in LUAD cells that overexpress NPC2 (**Figure 7B**). Thus, we proposed that NPC2 could influence the progression of LUAD through the inhibition of the PI3K/AKT signaling pathway. Rescue experiments demonstrated that both overexpression NPC2 and the PI3K activator 740 Y-P promoted the NPC2 mediated decreased proliferation, migration, and invasion capacity of LUAD cells (**Figures 7C, D**). These results indicated that NPC2 inhibits cell proliferation and migration/invasion by suppressing the AKT/mTOR signaling pathway in LUAD cells.

To investigate the roles of NPC2 in LUAD tumorigenesis *in vivo*, A549 cells with upregulated NPC2 were subcutaneously injected into BALB/c mice. The average volume and weight of tumors in the groups with NPC2 overexpression were significantly reduced compared to the control group (**Figures 7E–G**). Immunohistochemical (IHC) analysis revealed a notable reduction in Ki-67 expression in the xenograft tumor tissues of the NPC2 overexpression groups when juxtaposed with those from the control tumors (**Figure 7H**). These results indicated that overexpression NPC2 suppressed the growth of LUAD cells *in vivo*, which was consistent with the results *in vitro*.

### 3.7 Pharmacologic NPC2 overexpression enhances chemotherapy efficacy

**Figure 8:**
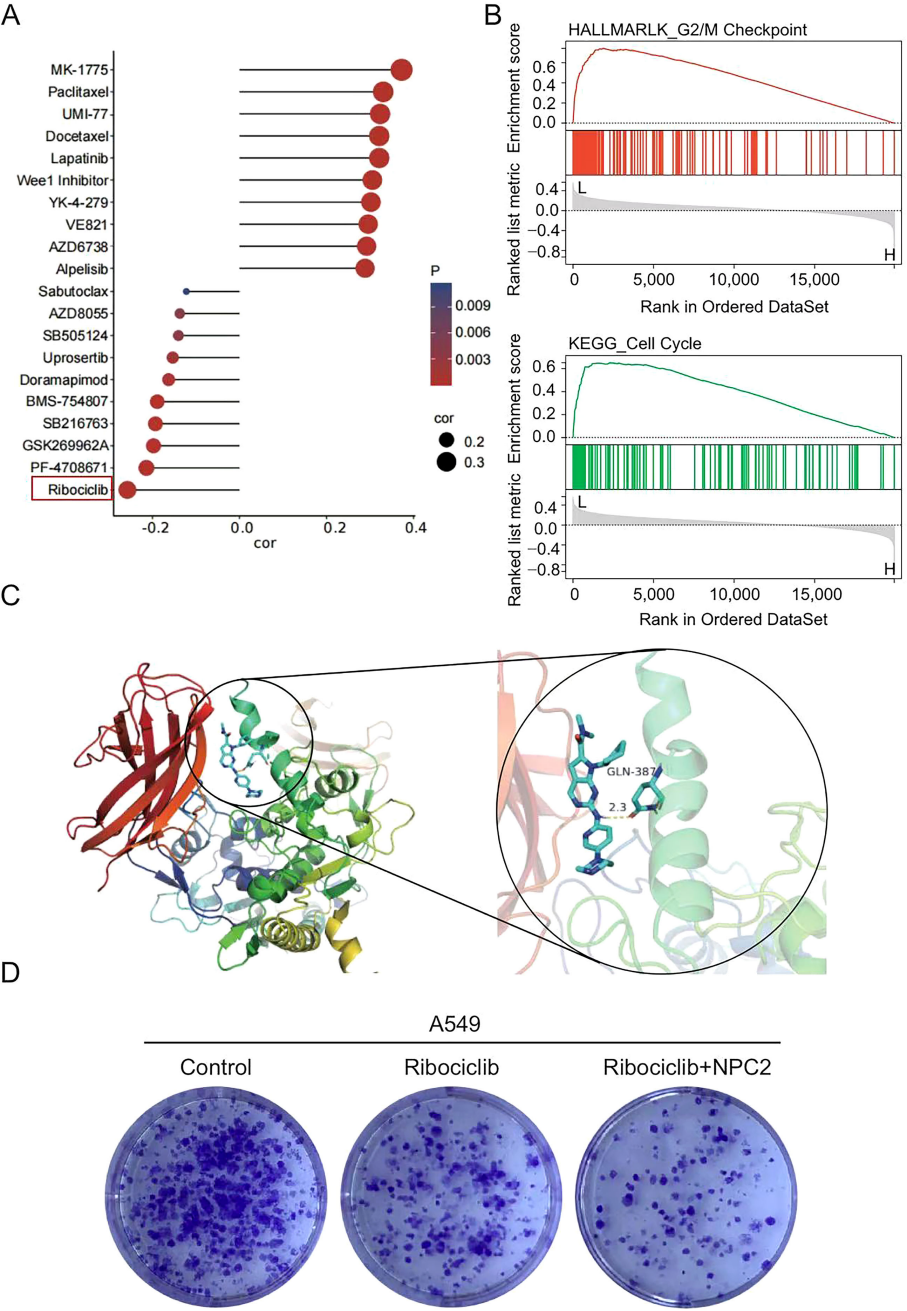
Pharmacologic NPC2 overexpression enhances chemotherapy efficacy. **(A)** Analyzing the correlation between the expression level of NPC2 and sensitivity to drugs. **(B)** GSEA analysis of the effect of NPC2 expression on cell cycle pathways. **(C)** Molecular docking analysis. **(D)** Proliferative ability of A549 cells was measured by colony formation assay.

The observed suppressive effects of NPC2 overexpression on LUAD led us to explore its therapeutic potential further. We initially performed a drug-correlation analysis against NPC2 expression. The expression of NPC2 mRNA is negatively correlated with Ribociclib sensitivity, meaning that as NPC2 expression increases, the IC50 of Ribociclib decreases (**Figure 8A**).Research shows that Ribociclib is a cyclin dependent kinase inhibitor. Subsequently, GSEA pathway analysis was conducted based on the HALLMARK and KEGG pathway gene sets, and the results showed that high expression of NPC2 was associated with inhibition of the cell cycle pathway (**Figure 8B**). To determine whether elevated levels of NPC2 enhance sensitivity to Ribociclib, further investigation through molecular docking analysis was prompted. The results from the molecular docking indicated a robust and stable interaction between Ribociclib and NPC2, featuring a binding energy of -7.8 kcal/mol. Notable docking sites were identified, with the residue GLN-387 involved in interactions through hydrogen bonding (**Figure 8C**).These findings indicating NPC2 potential as a therapeutic agent in modulating Ribociclib drug sensitivity in LUAD treatment. Finally, functional validation was undertaken using colony formation assays. These results suggest that NPC2 may potentiate the inhibitory effect of Ribociclib on LUAD cell proliferation (**Figure 8D**).These findings indicate that upregulation of NPC2 enhances sensitize LUAD cells to Ribociclib, supporting its potential as a therapeutic strategy for lung adenocarcinoma.

Additionally, to further elucidate the impact of NPC2 on immunotherapy, we analyzed data from the TCGA-LUAD, GSE13213, and GSE50081 datasets. The results of the ESTIMATE analysis indicated a positive correlation between NPC2 expression and ImmuneScore (*P* < 0.001, r = 0.347; *P* < 0.001, r = 0.425; *P* < 0.001, r = 0.191) (**Supplementary Figure S2A**). Utilizing the TIDE algorithm to predict potential immune responses, we found that NPC2 expression was significantly elevated in responding patients compared to non-responding patients (**Supplementary Figure S2B**). Meanwhile, NPC2 expression exhibited the strongest correlation with the infiltration of T follicular helper cells and mast cells (**Supplementary Figure S2C**). Single cell transcriptome analysis based on non-small cell lung cancer datasets (GSE131907 and EMTAB6149) showed that NPC2 is mainly expressed in monocytes/macrophages, epithelial and fibroblasts (**Supplementary Figure S2D**). Collectively, these findings provide preliminary support for the predictive significance of NPC2 in immune checkpoint inhibitor (ICI) immunotherapy. In the future, further *in vivo* and *in vitro* models are necessary to investigate the synergistic effects of NPC2 overexpression in conjunction with immune checkpoint inhibitors for the treatment of LUAD.

## 4 Discussion

Lung cancer remains the predominant cause of cancer-related deaths worldwide, with lung adenocarcinoma (LUAD) being the most common histological variant, making up roughly 50% of all cases of non-small cell lung cancer (NSCLC) (27, 28). Despite recent advancements in early diagnosis, molecular classification, and immunotherapy, the 5 year survival rate for LUAD patients remains low. Consequently, it is essential to clarify the molecular processes that drive the progression of LUAD in order to discover new biomarkers and therapeutic targets.

Gene expression is meticulously regulated by several epigenetic mechanisms, including methylation, mutation, and histone modification (29). Consequently, the integrated analysis of multi-omics data holds significant potential for screening tumor markers and identifying pathogenic targets, as well as facilitating early diagnosis and treatment (30). Currently, numerous studies have focused on the joint analysis of multiple omics. For instance, consensus immune-related lncRNA features, constructed through machine learning ensemble methods, can serve as indicators to evaluate prognosis, recurrence, and treatment benefits for colorectal cancer patients receiving fluorouracil-based ACT, bevacizumab, and pembrolizumab (31). Chen et al. employed LASSO analysis and 10-fold cross-validation to identify 22 image factors associated with bladder cancer, highlighting the potential clinical applications of machine learning in analyzing bladder cancer histopathological images (32). The application of random forests in machine learning to develop tumor microenvironment phenotypic signatures for breast cancer, based on the TME phenotypic characteristics of imageomics, can assist in predicting patients’ clinical responses to anti-PD-1/PD-L1 treatment (33). However, given that these prognostic models rely on expression profiles of mRNAs, miRNAs, or lncRNAs within particular pathways such as immunity, metabolic reprogramming, and m6A methylation, the utilization of data remains insufficient. Furthermore, the uniqueness and limitations of the selected modeling methods, coupled with the lack of rigorous validation in large multicenter cohorts, have hindered their widespread application in clinical settings.

TLRs play a crucial role as key innate immune sensors in identifying PAMPs and damage-associated molecular patterns (DAMPs) (34). TLRs regulate tumorigenesis by activating various immune cells (35). Recently, there has been increasing attention on the role and mechanisms of TLRs in the progression of specific cancers (36, 37), which has led to the development of various therapeutic strategies targeting TLRs. Several clinical trials have demonstrated that combination immunotherapy involving TLR agonists significantly enhances the treatment response in cancer patients (38, 39).

This study innovatively integrates mRNA expression profiles, DNA methylation data, and somatic mutation information related to the Toll-like receptor pathway, successfully establishing a comprehensive consensus subtype classification system for lung adenocarcinoma. Further analysis of subtype-specific differential expression was conducted, and an AIDPI scoring system was constructed using 101 machine learning algorithms. Clinical prognostic analysis indicates that high AIDPI scores are significantly correlated with shortened patient survival and advanced tumor progression. Additionally, we found that AIDPI is associated with the mutation status of classic tumor driver genes such as TP53 and EGFR. Previous studies have confirmed that mutations in these genes can enhance the sensitivity of lung adenocarcinoma to immunotherapy (40, 41). These research results not only enrich the molecular subtyping system of lung adenocarcinoma but also provide a scientific basis for optimizing precise stratified diagnosis and treatment strategies.

In addition to introducing AIDPI for the stratification of high-risk LUAD patients, we also identified NPC2 as a potential therapeutic target for this patient population. In recent years, multiple studies have revealed the significant role of abnormal cholesterol metabolism in the occurrence and development of tumors. The imbalance in cholesterol homeostasis is closely associated with the increased survival rates and inhibited apoptosis of malignant tumor cells (42), and it also significantly promotes tumor formation (43). In the cholesterol transport mechanism, NPC2 acts as a key protein that regulates the excretion and transport of cholesterol from lysosomes through its interaction with membrane proteins (44–46). Research has demonstrated that the activation of SREBP-1 can induce autophagy and increase NPC2 expression in cholesterol-depleted states, thereby facilitating lysosomal cholesterol release and accelerating glioblastoma progression (47). Notably, NPC2 effectively blocks the infiltration of stromal macrophage lines into early lung cancer tissues by regulating intracellular cholesterol balance and inhibiting CCL6 secretion (48). During liver cancer development, abnormal NPC2 expression regulates the proliferation, migration, and tumor formation of cancer cells by influencing the activation of the ERK1/2 signaling pathway (49). Beyond its role in tumor progression, NPC2 is also implicated in mechanisms of chemotherapy resistance. Research indicates that the downregulation of NPC2 expression leads to the accumulation of free cholesterol, which enhances the MAPK/AKT signaling pathway in hepatocellular carcinoma, thereby reducing the therapeutic efficacy of sorafenib (50). Furthermore, ALKBH5 plays a regulatory role in oxaliplatin resistance in colorectal cancer by inhibiting the YTHDF2-mediated m6A demethylation of NPC2 (51).However, the biological functions and associated molecular regulatory mechanisms of NPC2 in LUAD remain poorly understood. In this study, we demonstrated that elevated NPC2 expression correlates with favorable prognosis in the LUAD cohort. Furthermore, we discovered that the overexpression of NPC2 in LUAD cell lines results in decreased proliferation and enhanced apoptosis, which is attributed to the inhibition of the PI3K/Akt signaling pathway.

Cyclin-dependent kinases (CDKs) are essential for the regulation of the cell cycle (52). In recent years, Ribociclib, a targeted inhibitor of CDK4/6, has been developed and clinically evaluated. This therapy has notably extended progression-free survival and overall survival for patients battling advanced breast cancer (53, 54). However, despite the improved prognosis associated with CDK4/6 inhibitor treatment, approximately 10% of tumors exhibit initial resistance when used as monotherapy, resulting in a poor prognosis (55–57). Previous research has shown that combining Ribociclib with Alpelisib (PI3K inhibitor) yields a favorable synergistic effect in early breast cancer and colorectal cancer models (58, 59). Notably, our research suggests that NPC2, as a novel biomarker, can inhibit the growth of lung adenocarcinoma by suppressing the PI3K signaling pathway and enhance the sensitivity of Ribociclib in the treatment of lung adenocarcinoma. Furthermore, our single-cell analysis also found that NPC2 is mainly expressed in monocytes/macrophages, epithelial cells, and fibroblasts in the LUAD microenvironment. Previous studies have shown that based on ICD markers (60) and exhausted CD8^+^ T cell features (61), further integrated scRNA seq data to construct prognostic features through machine learning, both enhancing clinical prediction. Complementarily, spatial transcriptomics has revealed M2a-type TAMs as drivers of spread through air spaces via the CCL17-CCR4/β-catenin axis (62), suggesting precise therapeutic strategies for STAS-positive patients. Collectively, these studies contribute to our understanding of LUAD immunobiology and patient stratification.

Compared to earlier published studies, our research presents several significant differences. This study integrates omics information from all three dimensions of lung adenocarcinoma for the first time and employs ten clustering algorithms for a comprehensive analysis, effectively mitigating the influence of single algorithm preferences on the research findings. Additionally, the modeling factors derived from multi-queue prognostic gene screening considerably enhance the robustness and predictive performance of the model. Furthermore, by integrating data from four multicenter lung adenocarcinoma cohorts and combining them with ten machine learning algorithms, we constructed an AIDPI model aimed at minimizing the risk of overfitting. However, this study does have certain limitations. Firstly, despite employing calibration algorithms for data standardization, heterogeneity in sample size and sequencing platforms persists within the queue, affecting model performance. Therefore, further validation is required in a prospective, multicenter, and technically standardized cohort. Secondly, all samples were obtained from retrospective studies, and comprehensive validation of the AIDPI model’s clinical applicability is necessary through extensive prospective multicenter research. Lastly, the absence of certain clinical and molecular feature information in public datasets may impact the potential correlation analysis between AIDPI and related variables.

## 5 Conclusion

In summary, this study successfully established a prognostic evaluation model for lung adenocarcinoma by integrating Toll-like receptor signaling-related genes, employing bioinformatics analysis and machine learning algorithms. This research establishes a scientific basis for the creation of customized treatment strategies for LUAD patients with varying risk levels in clinical practice. Furthermore, we elucidated the carcinogenic mechanism of NPC2 in lung adenocarcinoma, confirming its potential as an effective biological indicator for the prognosis of LUAD patients. However, the specific mechanism of NPC2 in the process of LUAD metastasis requires further exploration using metastatic nude mouse models in future studies.

## Data Availability

The original contributions presented in the study are included in the article/**Supplementary Material**. Further inquiries can be directed to the corresponding authors.
