# Supplementary material for: Multi-omics integration and machine learning identify NPC2 as a prognostic and treatment-responsive regulator in lung adenocarcinoma
Source: Front Immunol. 2026 Jan 16;16:1697560. doi: 10.3389/fimmu.2025.1697560 (PMC12855401; doi:10.3389/fimmu.2025.1697560)
Supplement: Supplementary file 1 [file Supplementaryfile1.docx]

**Supplementary Figures**

**
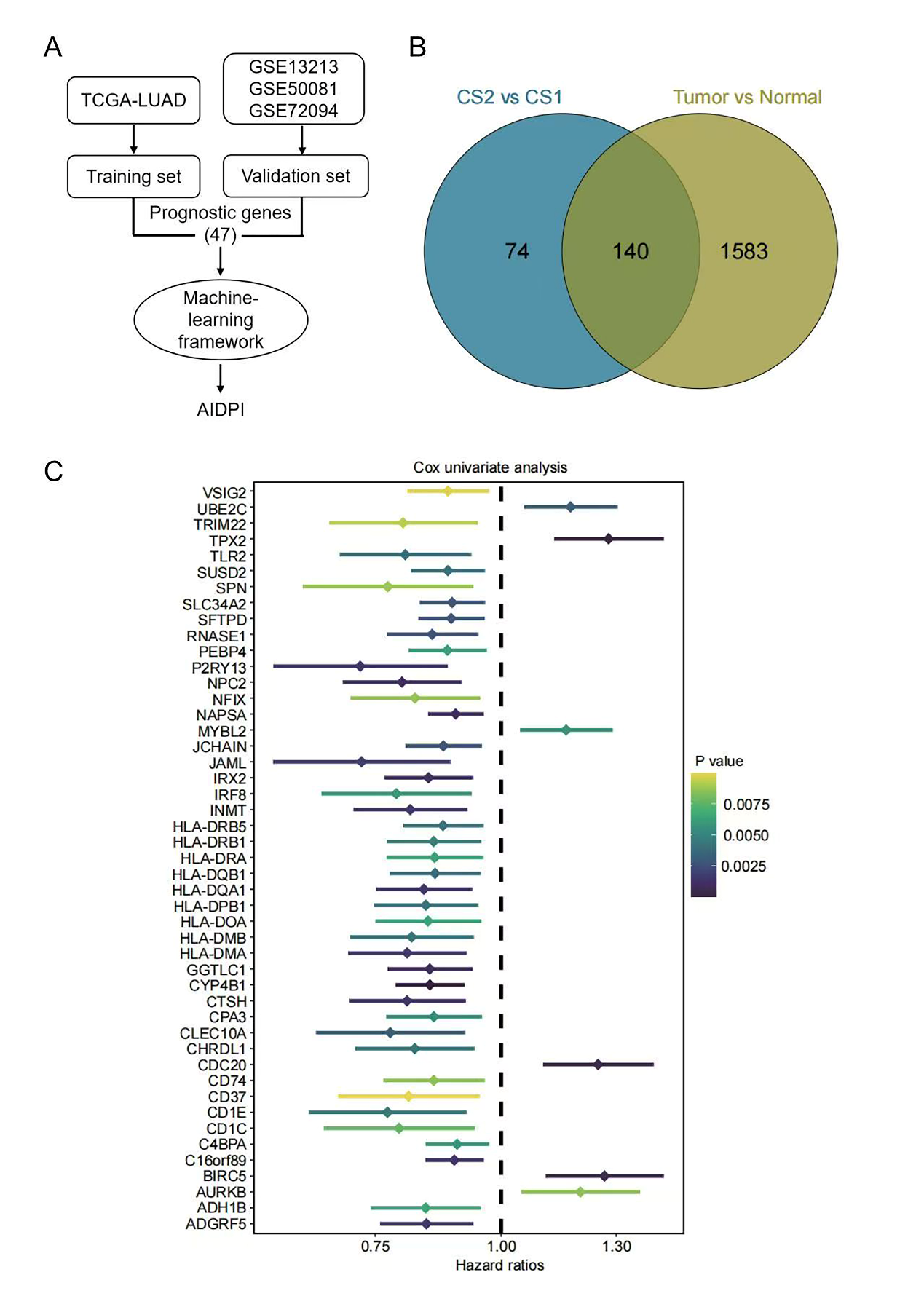
**

***Supplementary Figures 1.*** (A) Developed the AIDPI following the schematic. (B) Venn diagram displays intersecting differentially expressed genes. (C) Univariate Cox analysis identified 47 prognostic mRNA.


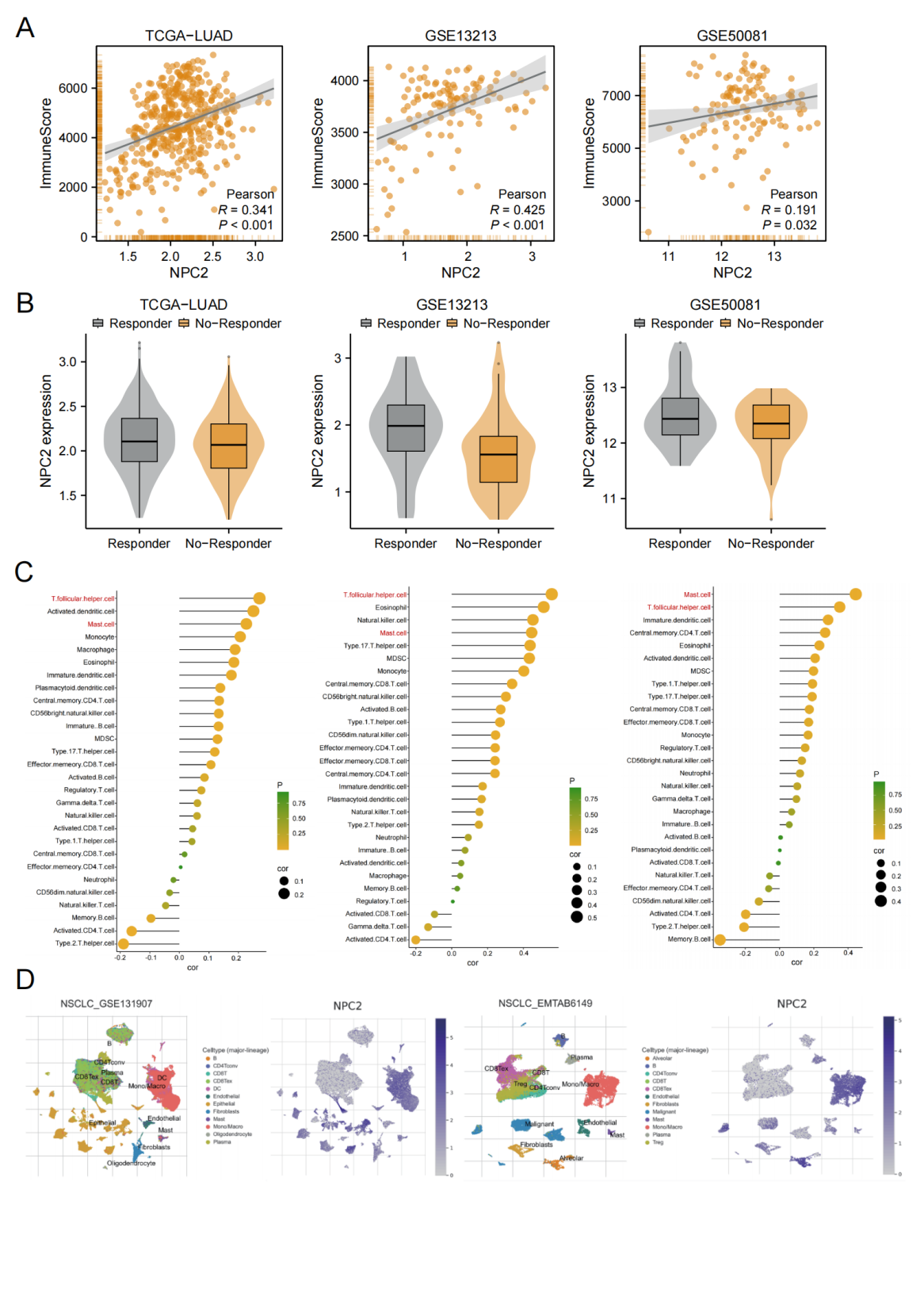


***Supplementary Figures 2*.** (A) Correlation analysis between NPC2 expression and ImmuneScore. (B) TIDE algorithm predicts the correlation between NPC2 expression and immune therapy response. (C) The correlation between NPC2 expression and immune cell infiltration. (D) NPC2 expression in NSCLC cells analyzed using single-cell transcriptomics from the TISCH2 database.
