# Supplementary material for: Multi-omics integration and machine learning identify NPC2 as a prognostic and treatment-responsive regulator in lung adenocarcinoma
Source: Front Immunol. 2026 Jan 16;16:1697560. doi: 10.3389/fimmu.2025.1697560 (PMC12855401; doi:10.3389/fimmu.2025.1697560)
Supplement: Supplementary file 2 [file Table1.docx]

**Supplementary Table**

**Supplementary Table 1*.*** **Primer sequence**

| Gene |  | Sequence (5'-3') |
| --- | --- | --- |
|  |  |  |
| NPC2 | Forward primer | TCCTGGCAGCTACATTCCTG |
|  | Reverse primer | ACAGAACCGCAGTCCTTGAAC |
| GAPDH | Forward primer | CTGGGCTACACTGAGCACC |
|  | Reverse primer | AAGTGGTCGTTGAGGGCAATG |
